# GCT: What happened after 10 years of curettage and cement? Retrospective study of 46 cases

**DOI:** 10.1590/1413-78522014220600973

**Published:** 2014

**Authors:** André Mathias Baptista, André Ferrari de França Camargo, Marcelo Tadeu Caiero, Daniel César Seguel Rebolledo, Luiz Filipe Marques Correia, Olavo Pires de Camargo

**Affiliations:** 1. Universidade de São Paulo, Faculdade de Medicina, Hospital das Clínicas, São Paulo, SP, Brasil, Institute of Orthopedics and Traumatology of Hospital das Clínicas da Faculdade de Medicina da Universidade de São Paulo. São Paulo, SP, Brasil

**Keywords:** Giant cell tumors, Bone neoplasms, Curettage, Process assessment (health care), Outcome assessment (health care)

## Abstract

**OBJECTIVE:**

To compare the functional outcome of patients with and without arthrosis, and to determine whether the development of arthrosis is related to the distance of the tumor from the subchondral bone.

**METHODS:**

Forty six patients treated for Giant-cell tumor (GCT) between 1975 and 1999 met inclusion criteria. GCT was diagnosed by percutaneous biopsy and confirmed after resection, in all cases. Campanacci's and Kellgren's classification, the distance of the cement to the articular surface and MSTS score were obtained throughout the sample.

**RESULTS:**

The distance of the cement to the subchondral bone was associated with greater risk of developing arthrosis, but there was no difference in MSTS scores between patients with or without arthrosis.

**CONCLUSION:**

We found that the distance from the cement to the subchondral bone has a prognostic value regarding future arthrosis, but it does not impact on the functional outcome.** Level of Evidence IV, Therapeutic Study**

## INTRODUCTION

Giant-cell tumor (GCT) of bone is a rare benign, but locally aggressive, primary bone tumor, accounting for approximately 5% of all primary bone lesions in adults. It comprises three cell types: mononuclear histiocytic cells, multinucleated giant cells (*osteoclasts-like*), and neoplastic stromal cells that are the main proliferating cell population. Etiology is still unknown.

On plain radiographs, the tumors appear as lytic lesions without matrix calcification and usually arise in the metaepiphyseal region of long bones. Campanacci described a radiologic classification as follows: (Table 1) Grade I lesions do not have cortical disruption and have a well-defined sclerotic medullary margin; Grade II lesions insufflate the bone, with cortex thinning, and have a well-defined non-sclerotic medullary margin; Grade III lesions have unclear margins, cortical disruption and soft tissue extension.^1^ This is a similar to the classification introduced by Enneking for benign bone tumors.^2^ Grade 2, or active lesions, are the most common form of presentation, accounting to aproximatelly 60% of cases. GCT lesions occur predominantly in the distal femur and the proximal tibia, but can ocurr anywhere in the skeleton.^3^ There is a slight predilection for females.^3^

**Table 1:** Campanacci's radiographic classification.1

Grade	Description
I	No cortical disruption and have a well-defined sclerotic medullary margin;
II	Bone insufflation, with cortex thinning, and a well-defined non-sclerotic medullary margin;
III	Unclear margins, cortical disruption and soft tissue extension.

Pulmonary metastasis occurs in approximately 1-3% of cases but there are reports of higher rates, such as 12.9%.^4^ In approximately 50% of cases, pulmonary metastasis occur after local recurrence in the distal radius.^5^ In such cases the mortality rate vary from 16-23%.^5^

The two most performed surgical procedures are intralesional resection (curettage) combined with high-speed burring and local adjuvance (phenol, liquid nitrogen, argon laser, electrocauterization), and *en-bloc* resection followed by reconstruction,^3^ but there is still no consensus regarding the choice of technique.^6^ Most surgeons use intralesional resection for Campanacci grades I and II, while in Campanacci III *en-bloc* resection followed by reconstruction is performed.^3^^,^ 7

Clinically they are locally aggressive and have a recurrence rate that vary widely from 0% to 65%. Classically the recurrence rates for primary treated lesions range from 0-18%.^3^ For recurrent lesions, the second recurrence rate is around 35% and is associated to Campanacci grade III lesions, pathologic fractures and intralesional resection.^3^

We agree with some authors that sometimes the functional outcome is better after intralesional resection than after *en-bloc* resection, even when a second procedure may be necessary (another intralesional resection in a recurrent lesion).^8^ For decades, GCTs have been treated in our institution mainly by intralesional resection, even in some Campanacci grade III lesions. Subchondral tumors also were treated in the same fashion, although some authors believe that when there is subchondral involvement, placing cement directly over the cartilage is harmful to it. Bone grafting between the cement and the cartilage is the recommended technique in these cases. However, it has been shown that there are no statistically difference in functional outcome when either cement or bone graft is used adjacent to the cartilage after curettage.^3^ Therefore, we hypothesized that: (1) after a follow-up of at least 10 years, radiographic arthrosis is more frequent in GCT lesions located less than 10mm of the subchondral bone when treated with intralesional resection (compared to GCT more than 10mm of the articular surface), and that (2) patients treated with intralesional resection that developed radiographic arthrosis in a follow-up of at least 10 years had no worse functional outcome than patients who did not develop arthrosis.

## PATIENTS AND METHODS

We conducted a cross-sectional, retrospective study based on the medical records of 190 patients treated for GCT at the Instituto de Ortopedia e Traumatologia of the University of São Paulo from 1975 to 1999. All patients were assessed for eligibility. 80 patients were initially excluded due to incomplete data or location in the axial skeleton; five patients were treated with a fibula graft, two had a simple curettage without cement, 11 had wide resection and reconstruction with prosthetic replacement, seven had wide resection without reconstruction, resulting in 85 patients that underwent intralesional resection + cement. Among these, 39 had a follow-up period of less than 10 years, resulting in a total of 46 cases that were included in this study. Inclusion and exclusion criteria are shown in Table 2.

**Table 2:** Inclusion and exclusion criteria.

Inclusion criteria	Exclusion criteria
Treatment by curettage, electrocauterization and cement;	Incomplete records Axial skeleton
Diagnosis of bone GCT confirmed by preop biopsy AND after surgery;	
> 10y of follow-up.	

**Figure 1:**
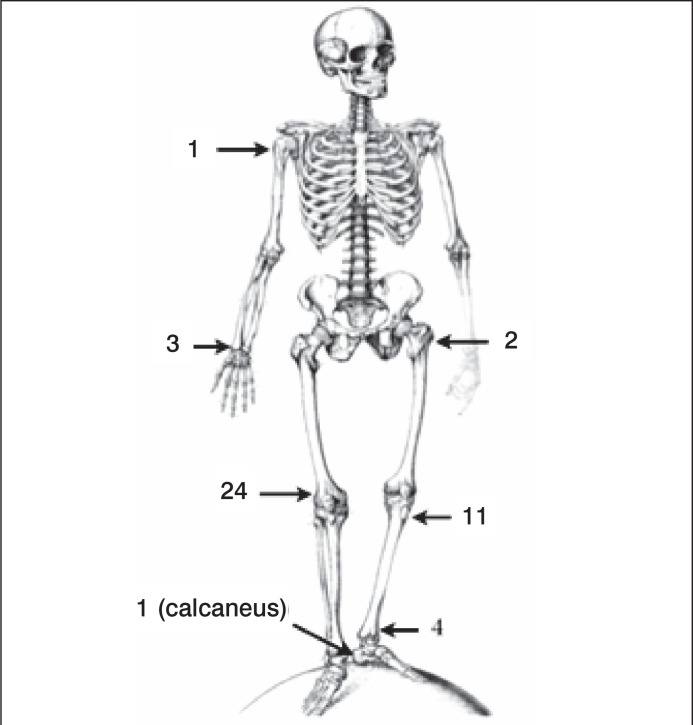
Anatomic location.

Clinical and pathologic data were obtained from hospital records and paraffin-embedded specimens. All patients underwent routine peripheral blood tests, radiographs, and biopsy, which was positive for GCT in all cases, and confirmed after surgery. All 46 cases were reviewed and confirmed by two pathologists (CRGCMO, RZF) with experience in musculoskeletal oncology. Data extracted from the charts included gender, age, anatomic location of the lesion, (Figure 1) Campanacci's bone destruction radiographic classification, Kellgren's arthrosis radiographic classification, distance of the cement to the articular surface in radiographs in millimeters, follow-up in years and MSTS functional score at the end of follow-up.

All the preoperative radiographs were independently evaluated by two of the authors (AMB, AFFC), classifying the bone lesions according to Campanacci^1^ in three grades, as described above. The same authors independently evaluated the immediate postoperative radiographs to measure the distance of the cement to the subchondral bone, in millimeters. The most recent radiograph of the last follow-up of each patient was also independently evaluated by the same authors to assess the presence of radiographic arthrosis using the Kellgren-Lawrence classification:^9^ Grade 0 - normal; Grade I: unlikely narrowing of the joint space, doubtful osteophyte; Grade II: small osteophytes, possible narrowing of the joint; Grade III: multiple, moderately sized osteophytes, definite joint space narrowing, some sclerotic areas, possible deformation of bone ends; Grade IV: multiple large osteophytes, severe joint space narrowing, marked sclerosis and definite bony end deformity. (Table 3) The Kellgren-Lawrence (K-L) classification is the most universally accepted method of classification of radiographic osteoarthritis.^10^ We decided to separate two groups (K-L < II or I and K-L ≥ II) to distinguish significant arthrosis. The MSTS score for each patient was obtained on the last follow-up visit.

**Table 3:** Kellgren-Lawrence classification.9

Grade	Description
0	Normal
I	Unlikely narrowing of the joint space, doubtful osteophyte
II	Small osteophytes, possible narrowing of the joint
III	Multiple, moderately sized osteophytes, definite joint space narrowing, some sclerotic areas, possible deformation of bone ends
IV	Multiple large osteophytes, severe joint space narrowing, marked sclerosis and definite bony end deformity

All demographic data and other qualitative variables were described using absolute and relative rates; quantitative variables were described using average values and standard deviation. The descriptive patients' demographics data are summarized in Table 4. It shows that the majority of the patients are female (60,9%), the average age was 31 years-old (SD = 12,8 years), the most common Campanacci grade was III (45,7%) and that the majority of the patients had less than 10mm of distance between the cement and the subchondral bone (73,9%). The most common affected location was the distal femur (52,2%) followed by the proximal tibia (23,9%) and distal tibia (8,7%). The average follow-up was 17,4 years (SD = 6,4). Most patients developed significant arthrosis (67,4%), here defined as Kellgren-Lawrence Grade II or grater, as described above.

**Table 4:** Descriptive patients demographic data.

Variable	Frequency	%
**Gender**		
Female	28	60.9
Male	18	39.1
**Age (years)**		
Average (SD)[range]	31.0 (12.8)[12-69]	
**Location**		
Distal femur	24	52.2
Proximal tibia	11	23.9
Distal tibia	4	8.7
Proximal femur	2	4.3
Distal radius	3	6.5
Proximal humerus	1	2.2
Calcaneus	1	2.2
**Campanacci grade**		
I	6	13
II	19	41.3
III	21	45.7
**Arthrosis (Kellgren grade)**		
0	15	32.6
I	9	19.6
II	4	8.7
III	15	32.6
IV	3	6.5
**Distance to articular surface**		
< 10mm	34	73.9
≥10mm	12	26.1
**Follow-up (years)**		
Average (SD)[range]	17.4 (6.4) [10-30]	
**MSTS (%)**		
Average (SD)[range]	80.6 (17.1) [40-100]	
Total	46	100

We verified the association of the qualitative characteristics with the presence of radiographic arthrosis using the *Chi-square test*, *Fisher exact test*, or the *likelihood ratio test*. Age, follow-up and functional MSTS scale were described as average values and standard deviation, and were compared using the Student-t test. All tests were conducted with a level of significance of 5%. This study was approved by the Scientific Committee of the IOT/HC/FMUSP, Protocol No 885/2011.

## RESULTS

The results are summarized in Table 5. There was no association of gender, age, Campanacci grade or the time of follow-up to the development of significant arthrosis (K-L ≥ II) (p > 0,05). 

**Table 5:** Results.

	Arthrosis (Kellgren)				Total	p
	No (0 – I)		Yes (II. III. IV)			
	n	%	n	%		
**Gender**						0.933
Female	9	32.1	19	67.9	28	
Male	6	33.3	12	66.7	18	
**Age**						0.876**
Average (SD)	31.4 (13.7)		38.8 (12.6)		31.0 (12.8)	
**Campanacci**						0.567#
I	3	50.0	3	50.0	6	
II	5	26.3	14	73.7	19	
III	7	33.3	14	66.7	21	
**Distance to subchondral bone **						<0.001*
< 10mm	4	11.8	30	88.2	34	
> 10mm	11	91.7	1	8.3	12	
**Follow-up (years)**						0.285**
Average (SD)	18.9 (7.2)		16.7 (5.9)		17.4 (6.4)	
**MSTS (%)**						0.921**
Average (SD)	80.2 (19.7)		80.7 (16.1)		80.6 (17.1)	
Total	15	32.6	31	67.4	46	

The distance of the cement to the subchondral bone, however, was associated with greater risk of developing significant arthrosis during follow-up (p < 0,001), but there was no significant difference in MSTS scores between patients with or without significant arthrosis (p > 0,05).

## DISCUSSION

GCT is a rare benign, however locally aggressive, bone tumor that may affect almost any bone, but is most common around the knee. The treatment modalities include intralesional resection (curettage) with local adjuvance, and *en-bloc* resection followed by reconstruction. Usually, the former is taken for Campanacci grades I and II, while the latter is performed in Campanacci grade III. Our institution has a long tradition of intralesional resection in GCT cases, even in some Campanacci III tumors.^11^^-^ 15 We had the clinical impression that the functional outcome may be better after intralesional resection than after *en-bloc* resection, even when the articular surface might be compromised during the surgery or the follow-up period. Therefore, we hypothesized that the patients with tumors located less than 10mm of the subchondral bone treated with intralesional resection + cement had more radiographic arthrosis than those with tumors located 10mm or more of the subchondral bone. We also hypothesized that the patients with radiographic arthrosis had no worse outcome then those without arthrosis.

The demographic data shown in our study is well aligned to the literature. The slight female predominance in previous studies was also found in our study (60,9%), as well as the peak incidence of age around 30 years old.^1^^,^ 3 ^,^ 16 ^,^ 17 The location of the tumors mainly around the knee followed the same pattern as has been previously described, the more frequent locations being the distal femur and the proximal tibia.^1^^,^ 3 ^,^ 16 ^,^ 17 We had no multicentric tumors in our series and the literature reports this as an extremely rare situation.^1^^,^ 3 ^,^ 16 The radiographic grading distribution, however, was slightly different than previous reports. Usually the most common grade is II, followed by III and I (53-70%, 25-47% and 3-5% respectively.^1^^,^ 3 ^,^ 16 ^,^ 18 In this study, on the other hand, the most comon grade was III (45,7%), followed by grade II (41,3%) and grade I (13%). This may have happened because our institution is a national reference for bone tumors and patients can take a few months from the first symptoms until the first evaluation by our team. During this long wait, the tumor may progress from grade II to grade III. Besides, orthopedic surgeons may feel less comfortable to treat aggressive lesions such grade III GCTs as they may sometimes resemble sarcomas, and these cases are more likely to be referred to our institution.

It has been suggested that the use of bone graft under the cartilage may prevent the harm that the cement may cause; however, there are no statistically difference in functional outcome when either cement or bone graft is used adjacent to the cartilage after curettage.^3^ Many surgeons share our clinical impression that function may be better after curettage than after en-bloc excision and prosthetic reconstruction, and there are a few publications reporting equal or even better functional outcome after intralesional resection when compared to *en-bloc* resection.^7^^,^ 17 ^,^ 19

To our knowledge, this is the first study to correlate arthrosis to the presence of cement adjacent to the articular cartilage, and the final functional outcome to the presence or absence of radiographic arthrosis.

One hypothetical consideration that can be made to justify our results is that the operated site may be denerved by the surgical procedure, and the subsequent arthrosis does not result in pain, just like a Charcot joint. There is no such scientific evidence, but it certainly makes sense.

We recognize limitations to our study. First, as the study spawned three decades, many different surgeons performed the operations. Thus, even with the general guidelines being followed, minor differences in surgical techniques may have existed. Second, as this is a rare disease, there was not a large number of patients, precluding any multivariate analysis that would control for potentially confounding variables. Third, we did not analyze other data such as complication rates or local recurrence. Forth, the only functional score used was the MSTS score, while there are many other scores more specific for each joint or location. The reason to use the MSTS score is the same reason we chose to use the Kellgren-Lawrence radiographic classification system for arthrosis: it is a universal score system for any location in the body.

## CONCLUSION

In conclusion, we found that the distance from the cement to the subchondral bone has prognostic value regarding future arthrosis, but it does not impact on the functional outcome.
